# Three-dimensional printing template for intraoperative localization of pulmonary nodules in the pleural cavity

**DOI:** 10.1016/j.xjtc.2022.10.003

**Published:** 2022-10-08

**Authors:** Hai Tang, Peng Yue, Ning Wei, Lei Zhang, Wenteng Hu, Weiyan Sun, Xiong Cao, Lixin Liu, Ruijiang Lin, Shangqing Xu, Chenhan Wang, Xiang Ma, Yunlang She, Minjie Ma, Chang Chen

**Affiliations:** a Department of Thoracic Surgery, Shanghai Pulmonary Hospital, Tongji University School of Medicine, Shanghai, China; b Shanghai Engineering Research Center of Lung Transplantation, Shanghai, China; c Department of Thoracic Surgery, The First Hospital of Lanzhou University, Lanzhou, Gansu Province, China; d The International Science and Technology Cooperation Base for Development and Application of Key Technologies in Thoracic Surgery, Lanzhou, Gansu Province, China; e Medical Quality Control Center in Thoracic Surgery, Lanzhou, Gansu Province, China

**Keywords:** flexible material, intraoperative localization, pulmonary nodules, three-dimensional printing, CT, computed tomography, 3D, 3-dimensional, TPU, thermoplastic polyurethanes, VATS, video-assisted thoracoscopic surgery

## Abstract

**Objectives:**

Localization of pulmonary nodules is challenging. However, traditional localization methods have high radiation doses and a high risk of complications. We developed a noninvasive 3-dimensional printing navigational template for intraoperative localization. It can reduce puncture-related complications and simplify the localization process. This study will verify the feasibility of this method.

**Methods:**

Patients with peripheral pulmonary nodules were included in this study. The computed tomography scan sequences were obtained to design a digital template model, which was then imported into a 3-dimensional printer to produce a physical navigational template. Finally, the navigational template is placed into the patient's pleural cavity for intraoperative localization. The precision of the nodule localization and associated complications were evaluated.

**Results:**

Twelve patients were finally included in this study. Intraoperative navigational template localization was used in all patients. The success rate of intraoperative nodule localization was 100%, and the median time of localization was 19.5 minutes (range, 16-23.5 minutes). The deviation median of the navigational template was 2.1 mm (range, 1.1-2.7 mm). Among the included patients, no significant complications occurred during intraoperative localization.

**Conclusions:**

The 3-dimensional printing template for intraoperative localization is feasible, will cause no trauma to the patient, and has acceptable accuracy for application in nodules localization. This navigational template greatly simplifies the localization process and may potentially break the dependence of percutaneous localization on computed tomography scanning.

**Figure:**
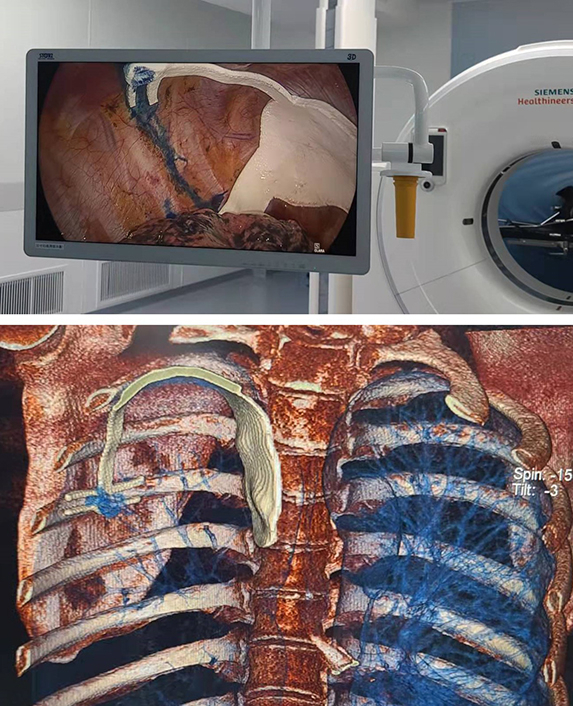
Intraoperative pulmonary nodule localization via a 3D printed flexible template.

**Central Message**

A 3D printed flexible template for intraoperative localization was designed and printed for pulmonary nodule localization and showed promising potential for clinical application.

**Perspective**

Localization of pulmonary nodules has become a challenge to thoracic surgeons since advanced CT technology is used for lung cancer screening programs. A 3D printed flexible navigational template for intraoperative localization could significantly facilitate the procedure of pulmonary nodule localization and alleviate possible puncture risks for patients.

With the popularization of lung cancer screening, the incidence of pulmonary nodules in thoracic surgery has risen sharply.^1^, ^2^, ^3^ However, pulmonary nodules are challenging to be located due to their small size and nonsolid features.^4^ Several methods of localization of pulmonary nodules are used to assist the surgeon in lung resection under video-assisted thoracic surgery (VATS).^5^^,^^6^ The most commonly used methods are percutaneous puncture with hookwire or microcoil, dye marking, and ethiodized oil injection.^7^, ^8^, ^9^, ^10^

However, it has been found that traditional localization has many disadvantages.^8^^,^^11^ First, the patient undergoes multiple computed tomography (CT) scans, increasing their radiation dose and economic burden.^12^, ^13^, ^14^ Then, the percutaneous puncture may increase the risk of complications, such as pneumothorax, hemothorax, and iatrogenic injury of large vessels.^15^ Next, preoperative localization requires the cooperation of the department of radiology and anesthesiology. The risk of hookwire shedding and pneumothorax will increase over time. Finally, it is challenging to complete the localization through the percutaneous puncture in patients with a thick chest wall. How can we avoid these problems? We propose a scheme for intraoperative localization of nodules: A navigational template is inserted into the pleural cavity during the operation to help the operator confirm the nodule's location.

Three-dimensional (3D) printing technology has been widely used in many fields, such as surgical planning, intraoperative guidance, and medical education.^16^^,^^17^ Also, 3D printing is one of the best techniques we can choose for producing customizable navigational templates. Several studies have designed and fabricated 3D printing devices using thoracic surface localization landmarks to guide the percutaneous localization of pulmonary nodules.^12^^,^^18^^,^^19^ However, the 3D printing materials currently used to produce medical devices are hard materials, such as polylactic acid and photosensitive resins. For pulmonary nodules localization in the pleural cavity, the hard material devices have many problems, such as inconvenient placement under VATS and damage to surrounding tissues and organs. To this end, we introduced thermoplastic polyurethanes (TPU) flexible material as the printing material of the navigational template. As a mature 3D printing flexible material, TPU is widely used in medical and health, engineering manufacturing, and many other fields due to its high tension and flexibility.^17^ Medical TPU material has no toxicity after contact with the human body and has reliable safety.^20^ Therefore, with the introduction of this flexible 3D printing material, the navigational template can be successfully implanted into the pleural cavity.

We designed a personalized and customized intraoperative localization device based on 3D printing, which can ensure the accurate localization of pulmonary nodules while reducing the incidence of puncture-related complications in patients. This study aims to evaluate the feasibility of the 3D printing navigational templates through clinical trials, providing a safe, accurate, and convenient nodule localization strategy (Video Abstract).

## Patients and Methods

### Study Population

This study included patients with peripheral pulmonary nodules between July 2021 and February 2022. The Ethics Board (Institutional Review Board) of the First Hospital of Lanzhou University approved this study (No. LDYYLL-2022-367, 01-07-2021). This study has been registered as a clinical trial (No. ChiCTR2100049139). All patients included in this study were pre-evaluated by imaging and thoracic surgeons. The surgical indication of pulmonary nodule resection followed the expert consensus of participating medical centers on diagnosing and treating early lung cancer.^21^ The inclusion criteria for patients were as follows: (1) age 18 years or more and less than 80 years; (2) peripheral pulmonary nodules identified by chest CT (nodules are located in the outer one-third of the lung field); (3) measured under the lung window of chest CT, the maximum diameter of the nodule was 20 mm or less; (4) the minimum distance from the edge of the completely solid nodular lesion to the pleura was 10 mm or greater; (5) according to the evaluation by the thoracic surgeon, the patient had indications for lung resection. If the inclusion criteria were met, patients were accurately informed of the risks and benefits of this study and signed an informed consent form. However, patients were excluded from the study if they met the following criteria: (1) adhesions in the thoracic cavity caused by any reason, which are not suitable for intraoperative pleural localization; (2) lack of preoperative CT scan sequences and cannot be obtained within the time limit; (3) the patient requested to withdraw from the trial. Once the patient was included in the study, the design and production of the navigational template were begun, and the entire process was completed on the same day. Subsequently, the prepared navigational template was sterilized for intraoperative use.

### Navigational Template Design and 3D Printing

**Figure 1:**
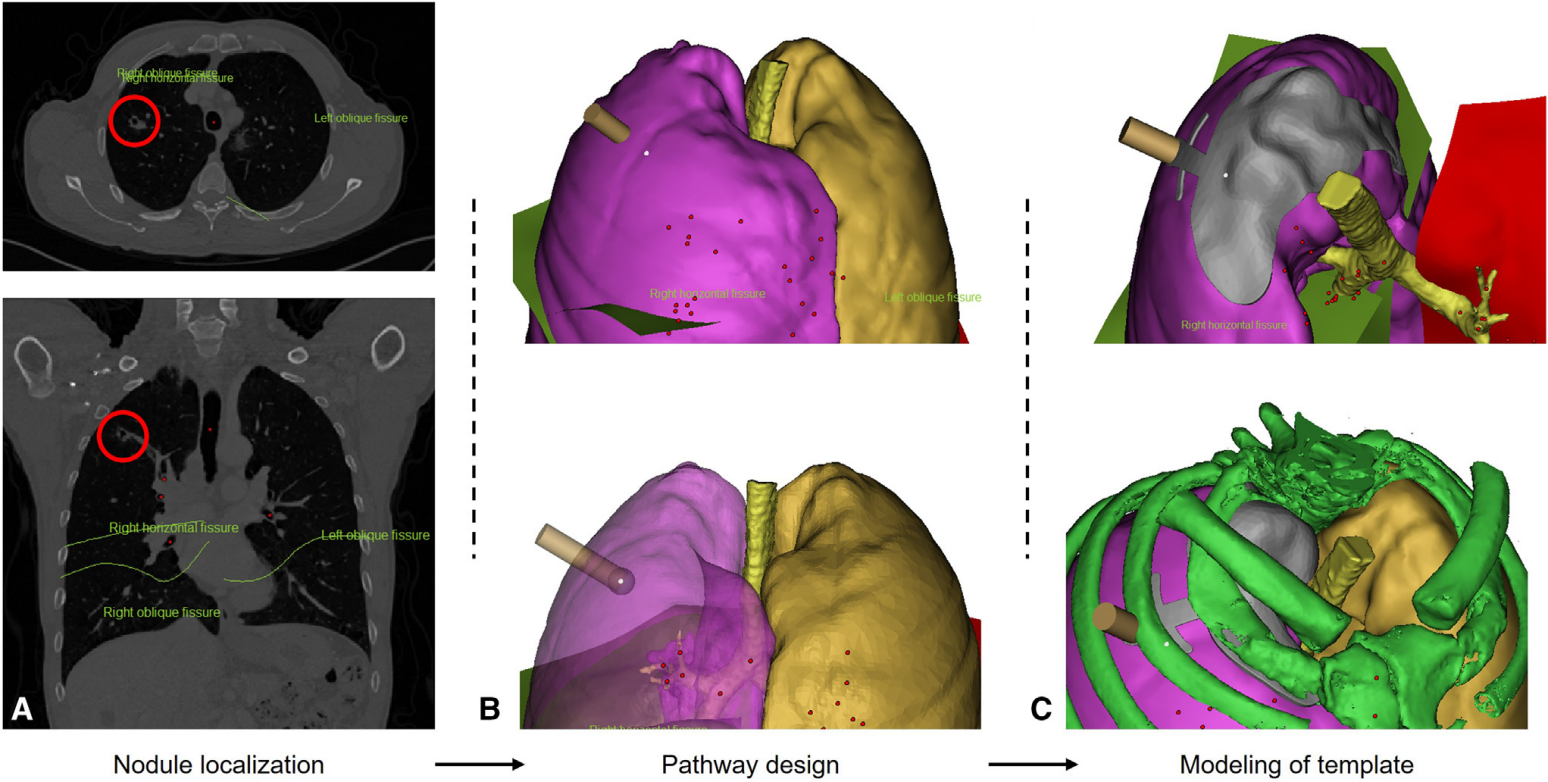
The scheme of template design. A, CT scan showing the location of the nodule in the patient (the *red circle* signified as the target nodule). B, Determining the projection of the nodule on the lung surface based on its location on the 3D modeling. C, Digital modeling of the navigational template.

**Figure E1:**
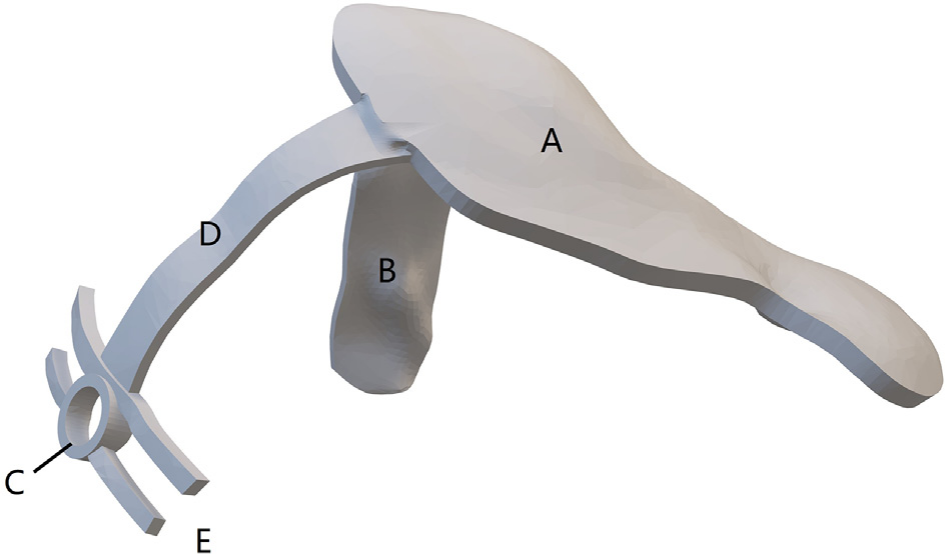
3D digital model of the navigational template. A, The key positioning module. B, The assisted positioning module. C, The ring positioning module. D, The arcuate positioning arm. E, The rib positioning module.

**Figure E2:**
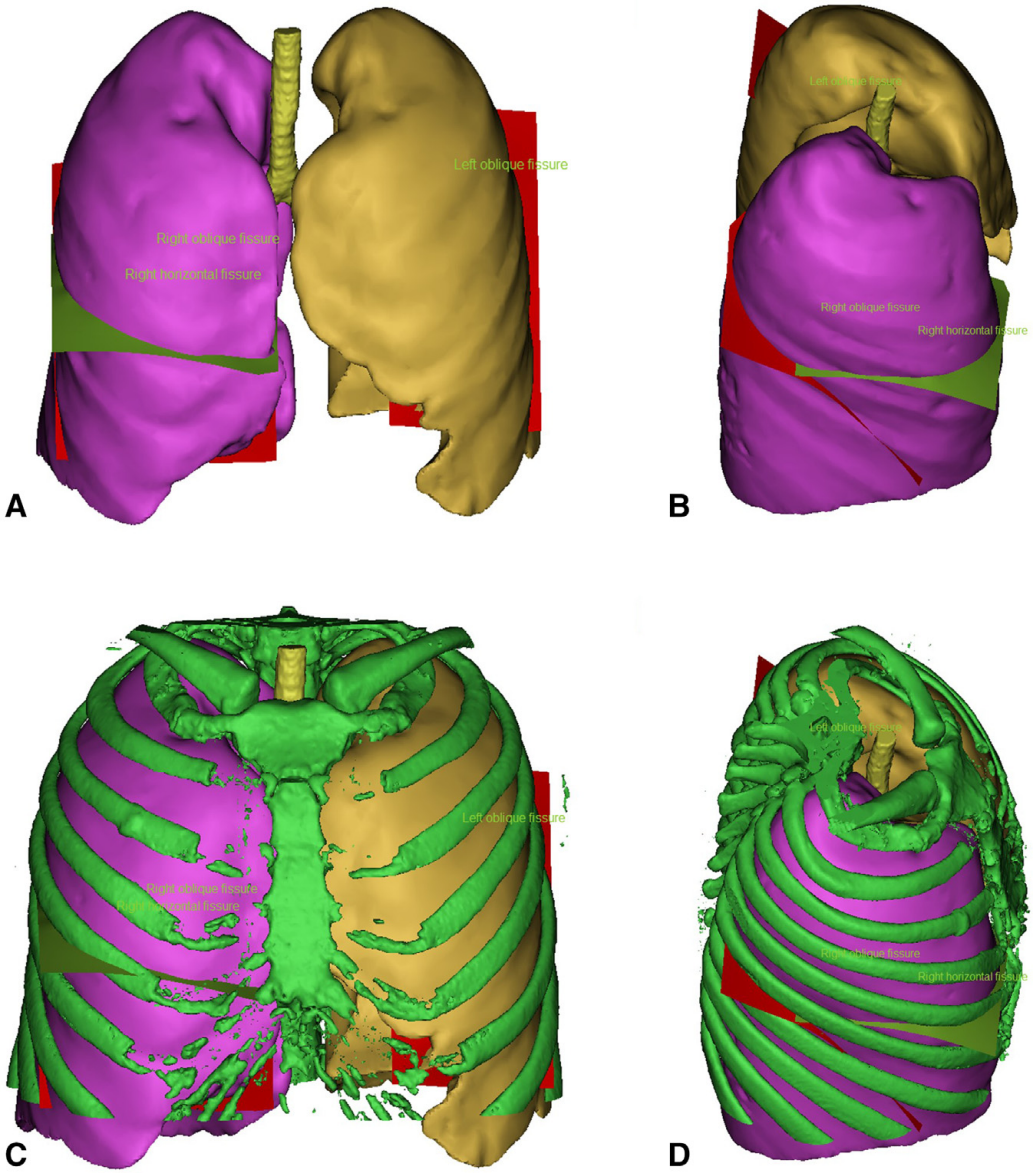
3D model construction of the patient's thorax. A and B, Front and side views of the 3D model of the thorax. C and D, Front and side views of the 3D model of the thorax with bony cage.

**Figure E3:**
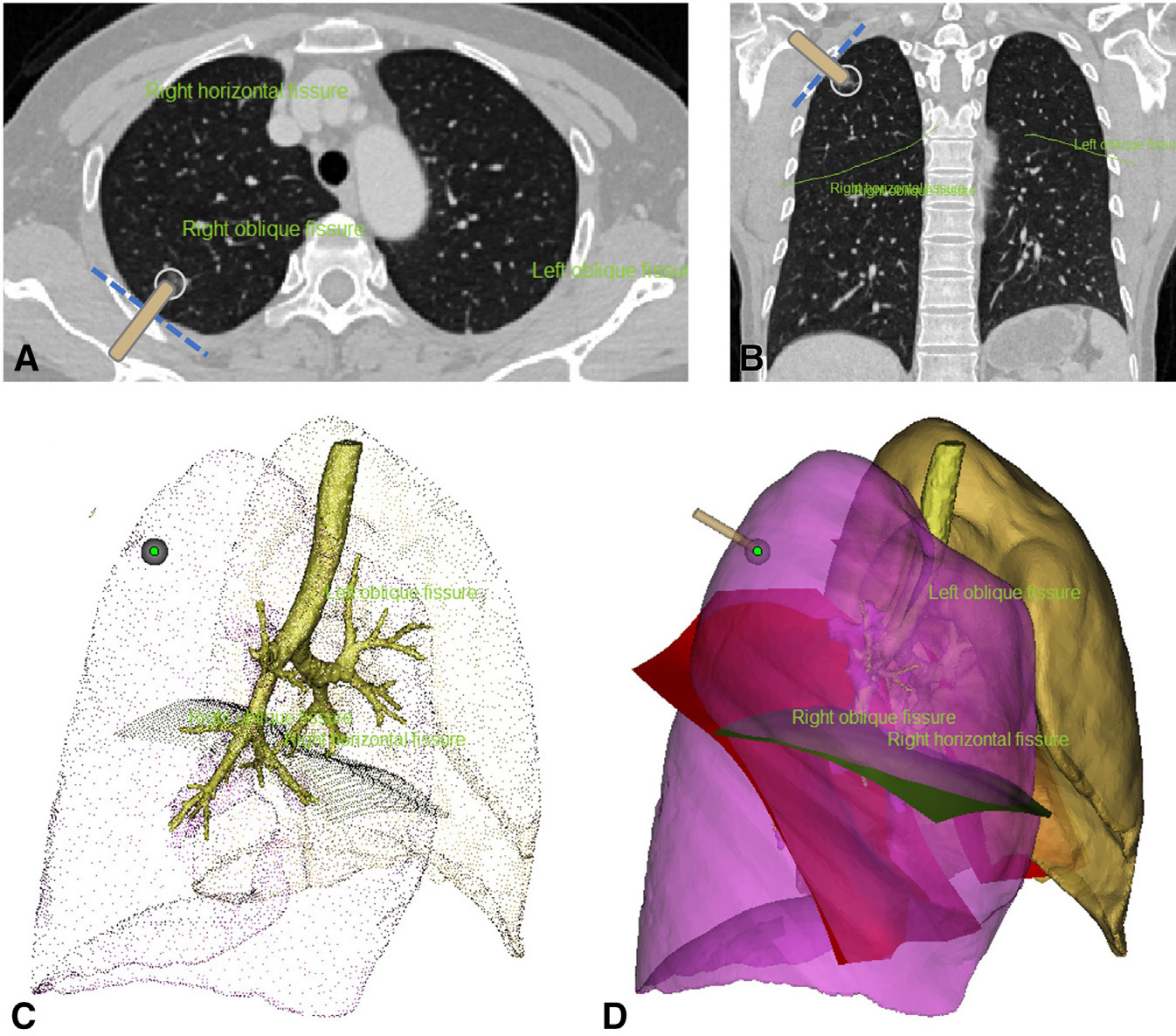
Determination of lung surface anchor points. A, Projection of the nodule on the lung surface in horizontal section. B, Projecting the nodule on the lung surface in the coronal section. C, Location of pulmonary nodules on the 3D model of the thorax. D, The designed inserting route shown in the 3D model of the second patient's thorax.

**Figure E4:**
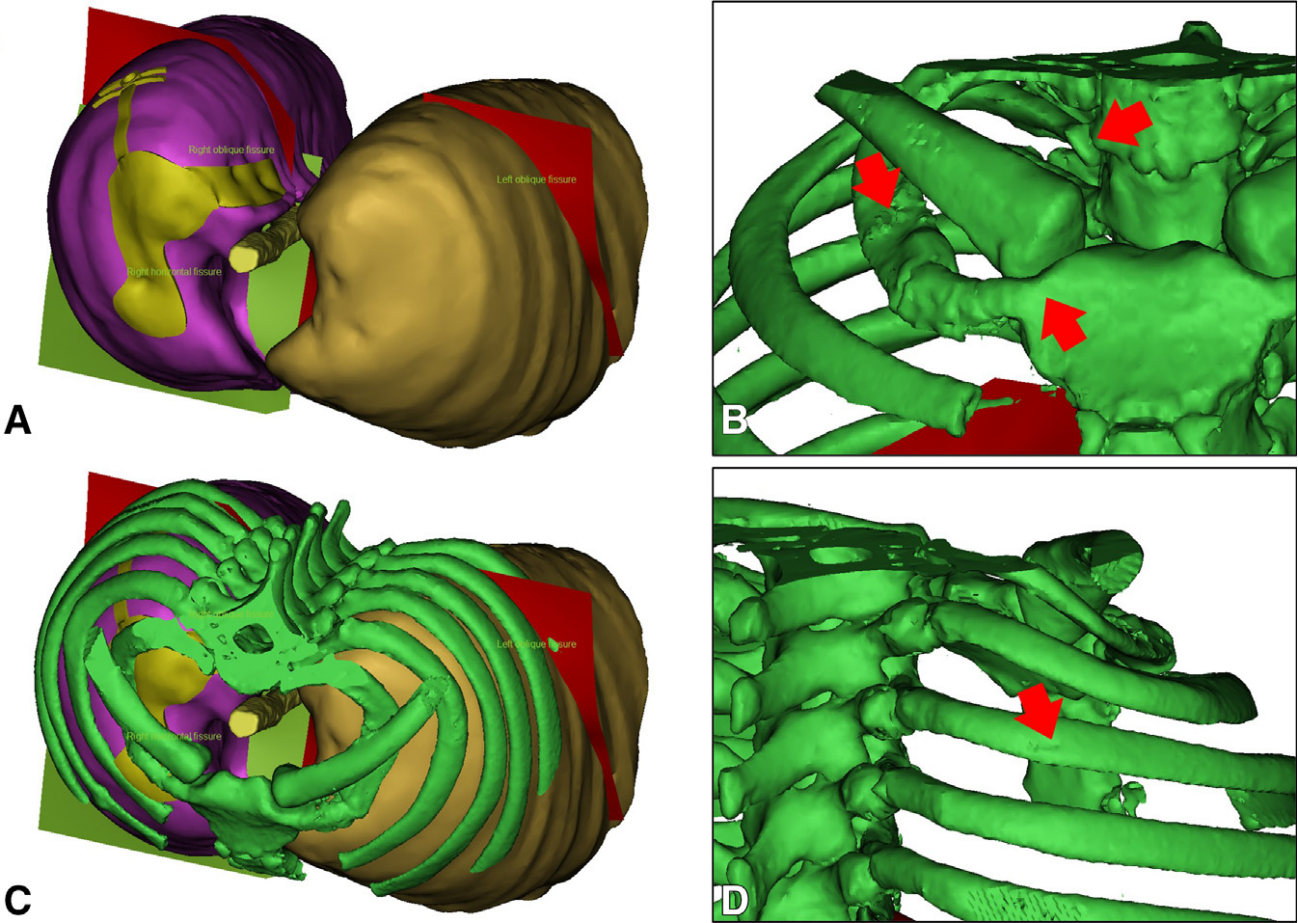
The anatomic landmarks selected to guide template placement. A, Position of the navigational template in the pleural cavity. B, The anatomic landmarks guide template placement (*red arrow*): first costal margin, sternoclavicular joint, and cost-vertebral joint. C, The relative position of the navigational template to the bony landmarks. D, Based on the position of the ribs (*red arrow*), determine the location of the navigational template.

**Figure E5:**
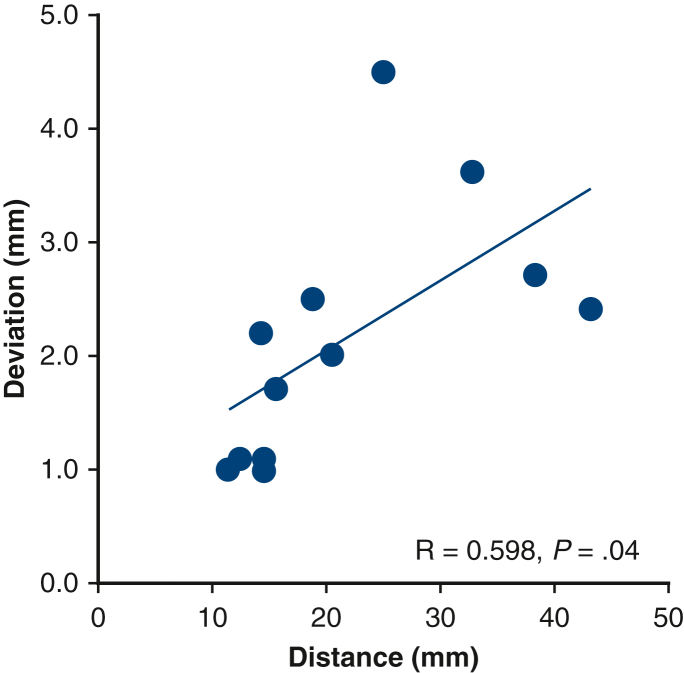
The correlation analysis between the nodule's distance from the pleura and the localization accuracy.

After the patients were included in the study, the CT scan sequences were obtained from Picture Archiving and Communication Systems. The First Hospital of Lanzhou University performed CT scans with a thickness of 1.5 mm on patients. After the sequence was acquired, the position information of the pleural cavity, bony landmarks (first costal margin, sternoclavicular joint, and cost-vertebral joint), and pulmonary nodules were determined. Subsequently, Mimics (Mimics Research 20.0, Materialise NV) software was used to process the acquired CT scan sequence information. First, we established a 3D digital model of the patient's chest and integrated the location information of the nodules into the digital model. We built a digital model of the navigational template based on the location information collected previously (Figure 1). The 3D model of the navigational template is shown in Figure E1. The navigational template consists of 5 functional modules: (1) The key positioning module uses the thoracic lung apex as the positioning site. This part is the anchor point of the navigational template, which assists the placement and position calibration of the navigational template during surgery (Figure E1, *A*); (2) the assisted positioning module uses the upper thoracic vertebra as the positioning part, which can assist in the alignment of the navigational template and undertake the role of fixation and support (Figure E1, *B*); (3) the ring positioning module is used to indicate the position, and the center of the ring corresponds to the projection of the pulmonary nodule on the lung surface (Figure E1, *C*); (4) the arcuate positioning arm is the connecting device between the key positioning module and the annular positioning module, which is consistent with the curvature of the thorax and can be fitted entirely with the thorax (Figure E1, *D*); (5) the rib positioning module verifies the navigational template again. When correctly placed, the position and direction of the rib positioning arm will be consistent with the rib (Figure E1, *E*). The detailed design steps of the navigational template are shown in Figure E2, Figure E3, Figure E4.

After the 3D digital model was established, we used the 3D printer (Black Flame Medical Technology) to print the physical model using the flexible printing material TPU (Lubrizol). Each navigational template costs between $30 and $50.

The digital model design of the navigational template takes an average of 45 minutes, whereas it takes 3 to 4 hours to print the physical template. The navigational template will then be sterilized by low-temperature plasma for intraoperative use.

### Localization Procedure

**Figure 2:**
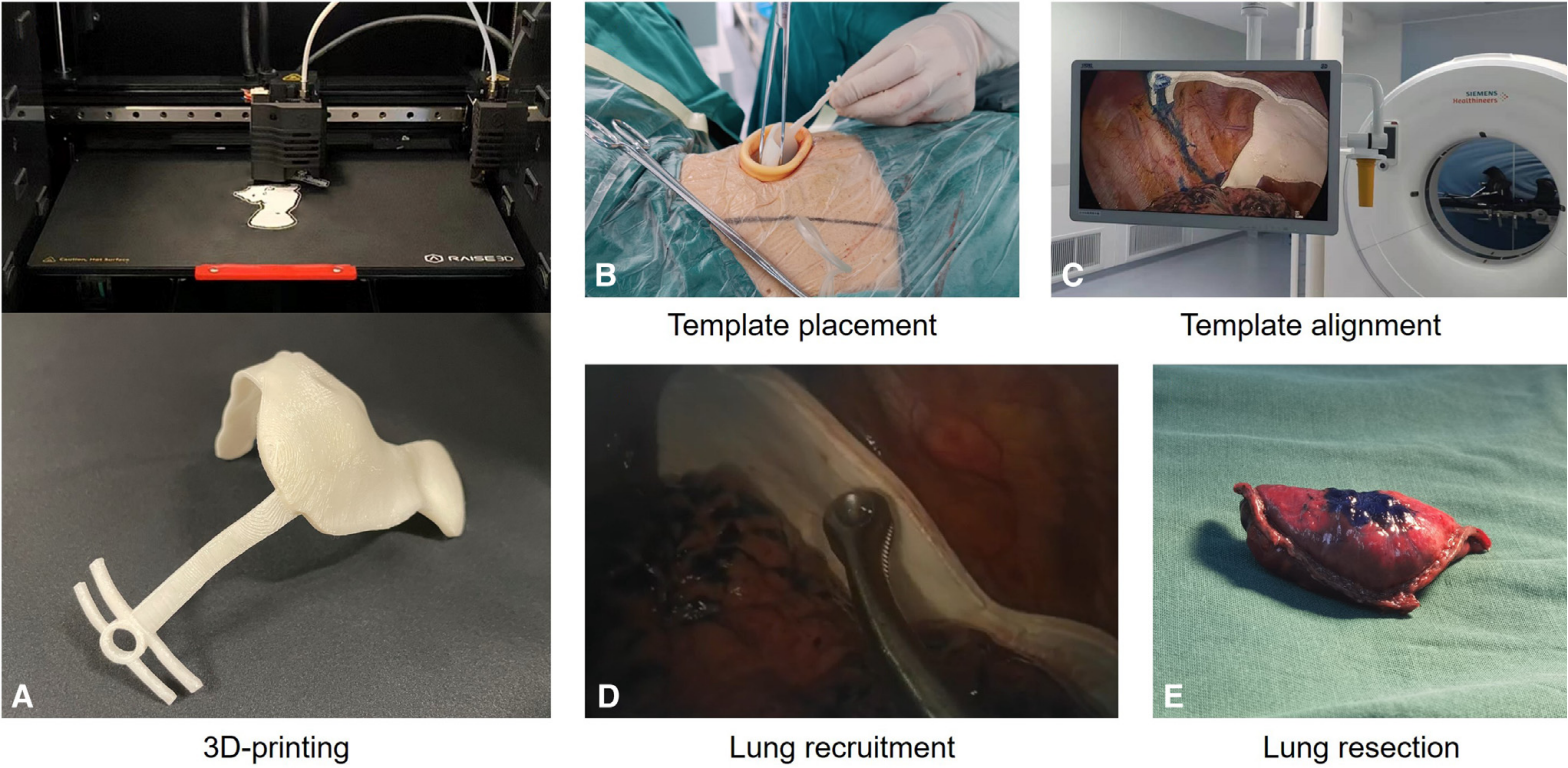
The process of pulmonary nodule localization using the navigational template. A, 3D printing of the navigational template. B, Placement of the navigational template in the pleural cavity. C, Alignment of the navigational template in the pleural cavity. D, Recruitment by negative pressure suction. E, Lung sample after lung resection.

After opening the intercostal incision, the anesthesiologist assists in collapsing the lung on the surgical site. Subsequently, the navigational template will be inserted into the pleural cavity from the incision of the VATS. The key positioning module of the navigational template will be placed on the top of the pleural cavity so that it matches the first rib, the spine, and the bulge of the sternoclavicular joint. Next, the accuracy will be verified according to the assisted positioning module and rib positioning module of the navigational template (Figure 2). Next, we prepare a cotton ball with methylene blue dye and fix it in the ring positioning module of the navigational template to mark the corresponding location on the lung surface.

**Figure 3:**
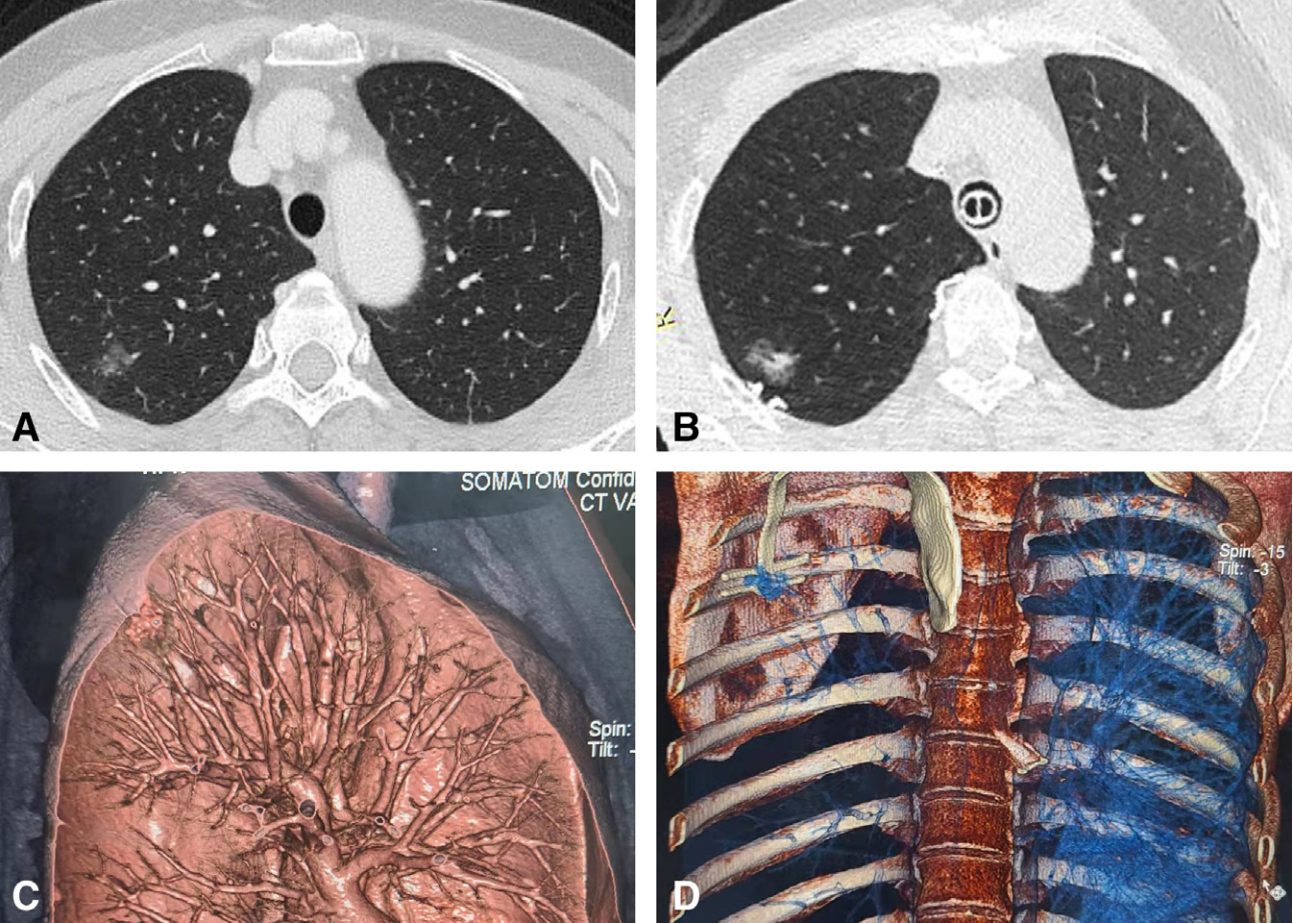
The precision of template-guided localization evaluated by intraoperative CT scanning. A, CT scan before template placement. B, Intraoperative CT scan after template placement. C, Location of nodules under 3D modeling. D, Location of the template under 3D modeling.

Subsequently, the anesthesiologist was asked to inflate the lungs. The ventilation tube was clamped on the surgical site at the end of total inflation. After the lung was fully inflated, an intraoperative CT scan in the hybrid operating room was performed (Figure 3). At the same time, the lung surface can be stained with methylene blue dye to assist the doctors in locating nodules. Details on the procedure for intraoperative localization using the navigational template are shown in the Video 1.

After the intraoperative CT scan, the lung will be collapsed again, and the navigational template was removed. After nodule resection, the resected specimens will be sent for frozen analysis. The related information will be recorded, including operation time, localization operation time, the distance between the lung surface design and the actual localization point, and the distance between the lesion and the design localization point. The deviation between the distance from the localization point center to the nodule center and the shortest distance from the nodule center to the pleura was used to indicate the localization accuracy of the navigational template. Adverse events during the operation were recorded.

### Surgical Procedure

For patients with solid nodules of invasive adenocarcinoma diagnosed by intraoperative frozen section, lobectomy, and systematic lymph node dissection are considered. Segmentectomy is performed if the patient's cardiopulmonary function cannot tolerate lobectomy or the patient has a strong desire to preserve lung function. Wedge resection and lymph node sampling are routinely performed for patients with adenocarcinoma in situ or minimally invasive adenocarcinoma.

### Statistical Analyses

Because this study is a small sample analysis, descriptive statistical analysis methods are mainly used. For continuous measurement, data were expressed as the median of the interquartile range and analyzed using the Mann–Whitney *U* test. For categorical count data, the Fisher exact test was used for analysis. SPSS statistical package software (SPSS Statistics 22.0, IBM) was used.

## Results

Between July 2021 and March 2022, we included 26 patients with indeterminate pulmonary nodules. Twenty patients agreed to participate in this study and signed the informed consent. Eight patients withdrew from the study because CT scan sequences were not obtained in time (n = 5), and the operation time was changed (n = 3). Finally, 12 patients completed the intraoperative navigational template localization.

The basic information and nodule characteristics of the patients are summarized in Table 1. Among all participants in this study, male patients accounted for 25%. The median age was 54.5 years (range, 38-58 years), and the median body mass index was 22.35 (range, 21.23-24.23). Three patients presented with pure ground-glass nodules, accounting for 25%, and 7 patients presented with solid nodules, accounting for 58.3%. The median nodule diameter was 13.25 mm, ranging from 10.75 mm to 18.00 mm, and the median distance from the outer edge of the nodule to the pleura was 17.25 mm, ranging from 7.25 to 30.93 mm.

**Table 1:** Clinical and radiologic characteristics of the population

Variable	n
Sex, % male	25% (3/12)
Median age, y	54.5 (38-58)
BMI	22.35 (21.23-24.23)
Nodule morphology	
Pure GGO	3
Mixed nodule	2
Solid nodule	7
Location	
RUL	6
RML	0
RLL	2
LUL	4
LLL	0
Nodule size, mm	13.25 (10.75-18.00)
Distance between the outer edge of nodule and pleura, mm	17.25 (14.35-30.93)

Age, BMI, nodule size, and the distance between the outer edge of the nodule and pleura were expressed as median (25th-75th percentile). *BMI,* Body mass index; *GGO,* ground-glass nodule; *RUL,* right upper lobe; *RML,* right middle lobe; *RLL,* right lower lobe; *LUL*, left upper lobe; *LLL,* left lower lobe.

All the included patients completed the localization during the operation. The median localization operation time was 19.5 minutes (range, 16-23.5 minutes). The deviation median was 2.1 mm, and the range was 1.1 to 2.7 mm. The deviation median of the upper/middle lobe and lower lobe lesions was 2.3 mm and 1.0 mm, respectively, but there was no statistically significant difference (*P* = .163) (Table 2). The correlation analysis showed no significant correlation (R = 0.598, *P* = .04) between the nodule's distance from the pleura and localization accuracy (Figure E5). It is worth mentioning that there were no complications related to the intrapleural navigational template during the procedure.

**Table 2:** Assessment of the precision of pulmonary nodule localization

Lesion	n	Time min	Deviation mm
All nodules	12	19.5 (16.0-23.5)	2.1 (1.1-2.7)
RUL + RML + LUL	10	19.5 (15.8-22.5)	2.3 (1.5-2.9)
RLL + LLL	2	27.0 (18.0 to NA)	1.0 (NA to NA)

*RUL,* Right upper lobe; *RML,* right middle lobe; *LUL*, left upper lobe; *RLL,* right lower lobe; *LLL,* left lower lobe.

After completing the nodule localization, the VATS lung resection will be performed. Intraoperative frozen-section analysis showed that no patient was diagnosed with adenocarcinoma in situ, 3 patients were diagnosed with minimally invasive adenocarcinoma, 5 patients were diagnosed with invasive adenocarcinoma, and 1 patient was diagnosed with atypical adenomatous hyperplasia. Three patients were diagnosed with benign nodules.

According to the frozen-section analysis, we further performed lobectomy for 1 patient and segmentectomy for 4 patients with invasive adenocarcinoma. Segmentectomy was performed because the patient's cardiopulmonary function was difficult to tolerate or they had a strong desire to preserve lung function, and the results showed lepidic predominant. All 5 patients had mediastinal lymph node dissection.

The median nodule diameter was 10.75 mm with a range of 7.50 to 15.38 mm, and the median distance from the incisal edge was 19.00 mm with a range of 16.25 to 22.50 mm (Table 3).

**Table 3:** Surgical approach and postoperative pathology

Variable	n
Surgical approach	
Wedge resection	6
Segmentectomy	4
Lobectomy	1
Pathology	
Benign	3
AAH	1
AIS	0
MIA	3
AD	5
Nodule size, mm	10.75 (7.50-15.38)
Margin distance, mm	19.00 (16.25-22.50)

Nodule size and margin distance were expressed as median (25th-75th percentile). *AAH,* Atypical adenomatous hyperplasia; *AIS,* adenocarcinoma in situ; *MIA,* minimally invasive adenocarcinoma; *AD,* invasive adenocarcinoma.

## Discussion

With the promotion of lung cancer screening, the detection rate of pulmonary nodules has increased.^22^^,^^23^ The localization of pulmonary nodules has been challenging, especially for nodules that are small in diameter without color change. In response to this problem, CT-guided localization was used to guide pulmonary resection. Although this method is widely used, there are still many limitations, including cumbersome procedures, high risk of complications, high radiation exposure, and accuracy limited by chest wall thickness. Because of these limitations, we designed a navigational template that the surgeon can use during surgery without puncture. It avoids the risk of complications caused by a puncture and simplifies the process of pulmonary nodule localization.

This navigational template can be placed directly on the top of the thoracic cavity, which is used as the anchoring site to indicate the projection of the pulmonary nodule on the visceral pleura. The surgeon can directly determine the nodule location with the nodule depth determined by the preoperative CT scan. In this study, we determined the accuracy of navigational template placement and indicated the location of pulmonary nodules. We can put it directly into the pleural cavity during the operation, avoiding punctures and multiple CT scans. It does not need to be positioned in the radiology department before entering the operating room, which creates convenience for patients and doctors.

Regarding the design of the anchoring site, first, localization in the pleural cavity avoids the localization failure caused by obscure anatomic landmarks due to excessive subcutaneous fat. However, there is also a large amount of soft tissue at the top of the thoracic cavity. Therefore, we chose bony landmarks (the first costal margin, sternoclavicular joint, and cost-vertebral joint) on the top of the pleural cavity as anchor points for the placement of the navigational template. At the same time, we designed localization marks parallel to the ribs around the localization points to provide feedback on the accurate placement of the navigational template. When the localization mark exactly matches the rib, the localization location is credible. Overall, the biases during the model design and template placement stages have been eliminated or minimized.

Considering the particularity of the operation site, we innovatively used flexible 3D printing material (TPU) as the material for the navigational template.^17^ Because the navigational template needs to be placed into the pleural cavity through the VATS surgical incision, which has requirements for its deformability and shape recovery ability. At the same time, the flexible material will not make the navigational template too sharp, avoiding damage to the organs during the placement process. In addition, this material has been widely used in the medical field and has reliable safety performance. Also, the cost per person for making the template was $30 to $50. Compared with traditional localization, this cost will significantly reduce the cost for patients. In terms of time cost, the intraoperative localization time is 20 to 30 minutes, which is close to CT localization but avoids the tedious process of preoperative localization.

In addition to using the navigation template to mark the locating points on the lung surface, we also introduced intraoperative CT to display the placement of the template in the thoracic cavity and double-check the accuracy of the placement of the template. Therefore, we performed this study in a hybrid operating room with intraoperative CT scan conditions. In the future, we may not require a hybrid operating room for fast and accurate intraoperative localization.

In this study, 12 patients had pulmonary nodule localization by navigational template and pulmonary resection was successfully performed. To our knowledge, this is the first study to apply a 3D printing navigational template to localization in the pleural cavity. We measured the deviation between the actual localization point of the navigational template and the designed localization point. Meanwhile, frozen sections of the specimen showed that the nodules were resected at the tumor-safe margins.

We discovered several factors that affect the accuracy of navigational template localization. The main reason is that when the pleural cavity is opened, the internal negative pressure disappears, and the lung collapses. It is impossible to fully inflate the lung only through the puffing of the lung. Moreover, under different pressures, the inflation degree is inconsistent, and the location of nodules will also change. Therefore, we sealed the incision after the navigational template was placed and then used a negative pressure suction device to create negative pressure in the pleural cavity to ensure complete lung inflation and accurate localization. The deviation of the upper/middle lobe nodules was relatively more significant than the lower lobe nodules, but the *P* value was .163 without statistical difference. Of course, this may also be due to accidental errors caused by the small sample size. Therefore, in our follow-up research, we will also explore the relationship between pressure and localization accuracy, which is expected to compensate for the inaccurate localization problem caused by incomplete lung recruitment.

Although this technique will leave a clear mark on the lung surface after 3D model localization, the resection depth cannot be directly indicated during the operation to avoid the risk of puncture as much as possible. The surgeon needs to decide the resection depth according to the preoperative CT scans. The results show that the success rate is 100%, which also shows that this method is feasible. This method is of sufficient reference value for clinicians.

Moreover, this method is not convenient enough, and it is easy to misuse and reduces the success rate of localization because the methylene blue dye is difficult to remove. We plan to improve the labeling device in the follow-up study (using the localization method first and labeling later). For example, after the navigation template is stably placed and the lung fully expands, methylene blue can be injected into the localization point through the reserved catheter.

### Study Limitations

Some limitations of this study need to be discussed. First, most nodules located by the navigational template were within 20 mm of the subpleural in this study. For deep pulmonary nodules, it is not easy to find the deep nodules through the projection of the visceral pleura after accurately locating the projection. Therefore, the effectiveness of the intraoperative navigational template for locating deep visceral subpleural nodules remains to be further verified. Second, because of the small number of participants in this study, the accuracy and safety of this application need to be evaluated in future randomized controlled trials. If the intraoperative navigational template proves to be as accurate as CT-guided localization, then direct intraoperative intrapleural localization can be achieved, freeing the reliance on CT scanning and percutaneous puncture.

## Conclusions

Constructing an intraoperative navigational template using 3D printing technology is feasible and has acceptable accuracy, as verified by intraoperative CT scans. Using this novel navigational template, we can facilitate and simplify locating nodules in patients, reduce patient trauma, and potentially even relieve the reliance on CT scans for percutaneous localization.

### Conflict of Interest Statement

The authors reported no conflicts of interest.

The *Journal* policy requires editors and reviewers to disclose conflicts of interest and to decline handling or reviewing manuscripts for which they may have a conflict of interest. The editors and reviewers of this article have no conflicts of interest.

## Supplementary Data

Video 1 The process of pulmonary nodule localization using the 3D printing navigational template. Video available at: https://www.jtcvs.org/article/S2666-2507(22)00526-0/fulltext.

Video Abstract Key results of 3D printing templates for intraoperative localization of pulmonary nodules. Video available at: https://www.jtcvs.org/article/S2666-2507(22)00526-0/fulltext.
