# Supplementary figures and images for: Three-dimensional printing template for intraoperative localization of pulmonary nodules in the pleural cavity
Source: JTCVS Tech. 2022 Oct 8;16:139–48. doi: 10.1016/j.xjtc.2022.10.003 (PMC9737054; doi:10.1016/j.xjtc.2022.10.003)

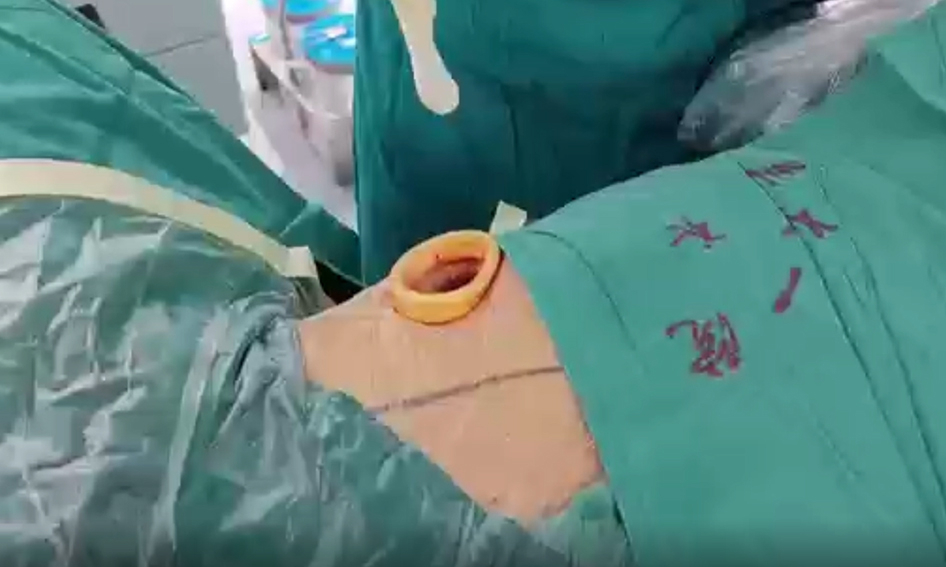

Supplement: Video 1 — The process of pulmonary nodule localization using the 3D printing navigational template. Video available at: https://www.jtcvs.org/article/S2666-2507(22)00526-0/fulltext. [file fx2.jpg]
